# Retinal axonal degeneration in Niemann–Pick type C disease

**DOI:** 10.1007/s00415-020-09796-2

**Published:** 2020-03-28

**Authors:** Joachim Havla, Marlene Moser, Clara Sztatecsny, Amelie S. Lotz-Havla, Esther M. Maier, Baccara Hizli, Regina Schinner, Tania Kümpfel, Michael Strupp, Tatiana Bremova-Ertl, Susanne A. Schneider

**Affiliations:** 1 Institute of Clinical Neuroimmunology, Ludwig-Maximilians University, Marchioninistr. 15, 81377 Munich, Germany; 2 Department of Neurology, Ludwig-Maximilians University München, Marchioninistr. 15, 81377 Munich, Germany; 3 Dr. von Hauner Children’s Hospital, Ludwig-Maximilians University, Munich, Germany; 4 Department of Neurology, University Hospital Bern, Inselspital, University of Bern, Bern, Switzerland; 5 Institute of Clinical Radiology, Ludwig-Maximilians University, Marchioninistr. 15, Munich, 81377 Germany

**Keywords:** Niemann–Pick type C, Heterozygosity, Clinical biomarker, Optical coherence tomography, Retinal neuroaxonal degeneration

## Abstract

**Objective:**

Niemann–Pick disease type C1 (NPC1) is a rare autosomal-recessive lysosomal storage disorder presenting with a broad clinical spectrum ranging from a severe infantile-onset neurovisceral disorder to late-onset neurodegenerative disease. Optical coherence tomography (OCT) is established to detect retinal degeneration in vivo. We examined NPC1-patients (NPC1-P), clinically asymptomatic NPC1-mutation carriers (NPC1-MC), and healthy controls (HC) to (1) identify retinal degeneration in NPC1-disease and (2) to investigate possible subclinical retinal degeneration in NPC1-MC.

**Methods:**

Fourteen NPC1-P, 17 NPC1-MC, and 31 age-matched HC were examined using spectral-domain OCT. Neurological examinations, clinical scales [modified Disability Rating Scale (mDRS); Scale for the Rating and Assessment of Ataxia (SARA); Spinocerebellar Ataxia Functional Index (SCAFI)], and video-oculography (VOG) were correlated with OCT data.

**Results:**

Macular retinal nerve fiber layer and volumes of combined ganglion cell and inner plexiform layer were significantly lower in NPC1-P compared to HC [mRNFL (µm):0.13 ± 0.01 vs. 0.14 ± 0.02; *p* = 0.01; GCIPL (mm^3^):0.60 ± 0.05 vs. 0.62 ± 0.04; *p* = 0.04]. No significant differences were found in NPC1-MC in comparison to HC. In NPC1-P, the amplitude of upward vertical saccades showed positive associations with peripapillary RNFL (*ρ* = 0.645; *p* < 0.05), and thinned GCIP (*ρ* = 0.609; *p* < 0.05), but not in NPC1-MC. In NPC1-P correlations between combined outer plexiform layer and outer nuclear layer (OPONL) with mDRS (*r* = − 0.617; *p* < 0.05) and GCIP with SARA (*r* = − 0.622; *p* < 0.05) were observed. Furthermore, in NPC1-MC, motor scores were negatively associated with pRNFL (*ρ* = − 0.677;* p* < 0.01).

**Conclusions:**

Using OCT, we showed retinal degeneration in NPC1-P and significant correlation between retinal neuroaxonal degeneration with clinical measurements. We observed a non-significant trend of retinal degeneration in NPC1-MC correlating with subclinical motor abnormalities. Based on these preliminary data, OCT may be an important marker of neurodegeneration in NPC1-disease after onset of clinical symptoms.

## Introduction

Niemann–Pick disease type C (NPC) is an autosomal recessively inherited neurovisceral lysosomal disorder caused by mutations in the *NPC1* or *NPC2* gene [1, 2]. The clinical phenotype ranges from an infancy-onset progressive, fatal disorder to an adult-onset, chronic neurodegenerative disease with heterogeneous clinical symptoms such as cognitive impairment, cerebellar symptoms, dystonia, vertical supranuclear saccade and gaze palsy, psychiatric disorders, and, less frequently, epilepsy [3]. Optic nerve pallor and perimacular gray discoloration are observed ophthalmologically, as well as histologically [4]. However, markers reflecting disease progression in NPC are not well established. Pathophysiologically, NPC is characterized by abnormalities of intracellular transport of endocytosed cholesterol and further lipids with their sequestration in lysosomes and late endosomes [5, 6]. Notably, dysregulation of brain cholesterol homeostasis is also present in some of the common neurodegenerative central nerve system (CNS) disorders such as Alzheimer`s disease (AD) and Parkinson’s disease (PD) [7–9]. In addition, NPC and AD share common pathophysiological mechanisms such as neurofibrillary tangle formation, increased amyloidogenic amyloid precursor protein (APP), early development of endosome abnormalities, and neuronal death [10–13]. Optical coherence tomography (OCT) is a non-invasive, cost-effective, and widely used imaging technique of the retinal layers introduced in the last few years as a potential marker of neurodegeneration in various neuroinflammatory and neurodegenerative disorders [14, 15]. Furthermore, in metabolic diseases such as Gaucher's disease (GD), which shows pathophysiological similarities to NPC [16], retinal neuroaxonal degeneration can be measured with OCT [17]. To our knowledge, OCT has not been used to study possible neurodegeneration in NPC yet.

In this study, we examined NPC1-P, clinically healthy NPC1-MC, and HC using OCT to (i) identify retinal degeneration in NPC disease using a non-invasive OCT technique and (ii) to investigate for possible subclinical retinal atrophy in NPC1-mutation carriers (MC). We aimed to show a correlation between OCT examinations and the clinically recorded scores to establish neuroaxonal degeneration as a disease-monitoring tool in NPC.

## Materials and methods

### Study population

Twelve NPC1-P with biallelic *NPC1* gene mutations and two patients with one identifiable mutation, a classical or variant filipin test in cultured fibroblasts and a typical NPC phenotype (vertical supranuclear saccade palsy, cerebellar ataxia, dysarthria, cognitive impairment, dystonia; mean age 24 [range 9–38]; 8 male) were recruited from the university hospital specialized unit (**Table ****1**). Mean duration of the disease was 14 ± 6.5 (SD) years. Seventeen of their asymptomatic first-degree family members, all confirmed heterozygous NPC1-MC (i.e., carrying one muted allele; mean age 50 [range 21–65]; 8 males) were included (**Table ****2**). No neurological comorbidity was diagnosed based on a detailed examination by experts in movement disorders (TBE and SAS). The motor examinations were not evaluated blinded. Two HC groups age-matched for patients and carriers were also enrolled (14 HC for NPC1-P: mean age 27 [range 12–38] 7 males; 17 HC for NPC1-MC: mean age 51 [range 21–66]; 9 male). In the NPC1-P group, only 26 of the 28 eyes could be examined. Two patients discontinued the examination incompletely after examination of the first eye. Eight out of fourteen patients suffered from an organic psychiatric disorder due to NPC disease and took antipsychotic drugs. To our knowledge, there is no evidence of an effect of psychiatric medication on the OCT data shown here. This also applies to the anticonvulsive drugs. Subjects, and single eyes, respectively, with concomitant potentially OCT-confounding diseases (glaucoma, diabetes mellitus, retinal surgery, retinal disease, and ametropia > 6 diopters) were also excluded. Evaluation criteria for this study were retinal thickness/volume and visual disability measured by high and low contrast visual acuity (VA). Patients and mutation carriers underwent neurological examination and demographic information was obtained as summarized in Tables 1 and 2. Other causes that could explain motor anomalies were excluded by anamnesis and examinations as needed. Written informed consent was obtained from all patients participating in the study. The study was performed in accordance with the Helsinki II Declaration and approved by the ethics committee of the Ludwig-Maximilians-University, Munich, Medical Faculty (part of project no 280-16). All participants (or their legal representatives) gave written informed consent.

**Table 1:** Demographic characteristics and clinical scales of NPC1-P

ID	Genotype *(NPC1)*	Age at OCT	Disease duration in years	mDRS	SARA	PV vertical saccades (Mean), (°/s)	PV horizontal saccades (Mean), (°/s)	Amplitude vertical saccades (Mean), (°)	Amplitude horizontal saccades (Mean), (°)	Duration vertical saccades (Mean), (s)	Duration horizontal saccades (Mean), (s)	Gain vertical smooth pursuit (Mean)	Gain horizontal smooth pursuit (Mean)
NPC1-P 01	c.709C>T (p.P237S) / N.y.f	30	25	5	6.5	185.9	410.7	15.3	25.4	0.46	0.34	0.65	0.76
NPC1-P 02	c 3246-5_3246-7del/c 3246-5_3246-7del^a^	20	10	7	7.0	46.8	514.7	14.3	30.1	0.80	0.24	0.53	0.74
NPC1-P 03	c.2474A>G (p.Y825C)/c.3160G>A (p.A1054T)	27	20	8	10.5	44.1	271.4	11.4	30.8	1.05	0.71	0.32	0.71
NPC1-P 04	c.2660C>T (p.P887L) /c.3019C>G (p.P1007A)	28	6	8	8.5	36.7	289.1	18.3	29.5	1.32	0.29	0.71	0.84
NPC1-P 05	c.2660C>T (p.P887L) /c.3019C>G (p.P1007A)	38	25	12	13.0	29.7	59.6	5.9	27.7	1.22	1.14	0.16	0.82
NPC1-P 06	c.2776G>A (p.A926T) /c.2861C>T (p.S954L)	35	20	13	12.0	22.5	71.5	4.4	26.6	0.90	1.29	0.20	0.56
NPC1-P 07	c.2195insT/ c.2474A>G (p.Y825C)	27	25	9	6.5	52.9	526.1	13.9	29.7	0.74	0.28	0.57	0.85
NPC1-P 08	c.1723delG/c.2861C>T (p.S954L)	27	12	12	12.5	60.2	376.9	15.6	29.6	0.70	0.25	0.56	0.79
NPC1-P 09	c.2974G>T (p.G992W) /N.y.f	15	7	10	10.0	30.1	290.4	9.9	26.1	2.14	0.17	0.36	0.67
NPC1-P 10	c.1211G>A (p.R404Q) /c.1843C>T (p.R615C)	21	11	18	19.0	49.1	275.1	6.4	28.3	0.44	0.40	0.37	0.70
NPC1-P 11	c.3246-25A>G/ c.3246-25A>G^b^	23	14	8	8.5	48.6	286.9	7.4	21.8	0.68	0.15	0.66	0.78
NPC1-P 12	c.3246-25A>G/ c.3246-25A>G^b^	27	10	8	13.5	77.5	148.3	9.9	24.8	0.72	0.37	0.54	0.72
NPC1-P 13	c.2780C>T (p.A927V) /c.3010 T>C (p.S1004P)	11	6	12	10	nd	nd	nd	nd	nd	nd	nd	nd
NPC1-P 14	c.2780C>T (p.A927V) /c.3010 T>C (p.S1004P)	9	4	3	3	nd	nd	nd	nd	nd	nd	nd	nd

*NPC1-P* NPC1-patients, *nd* no data, *N.y.f.* not yet found, *na* not applicable; ; *mDRS* modified disability rating scale, *SARA* scale for the assessment and rating of ataxia, *PV* peak velocity, *SD* standard deviation; characteristics of vertical saccades in response to 20° stimulus and horizontal saccades in response to 30° are listed

^a^An intronic variant not previously described in the literature (class 3), leading to skipping of exon 22

^b^An intronic variant not previously described in the literature, likely pathogenic based on prediction analysis

**Table 2:** Demographic characteristics and clinical scales of NPC1-MC

ID	Heterozygote genotype (NPC1)	Age at OCT	Motor score	PV vertical saccades (Mean), [°/s]	PV horizontal saccades (Mean), [°/s]	Amplitude vertical saccades (Mean), [°]	Amplitude horizontal saccades (Mean), [°]	Duration vertical saccades (Mean) [s]	Duration horizontal saccades (Mean), [s]
NPC1-MC 01	c.2861C>T (p.S954L)	64	2	368.7	476.82	18.39	26.56	0.18	0.14
NPC1-MC 02	c.2861C>T (p.S954L)	55	0	399.6	402.75	17.59	31.36	0.16	0.19
NPC1-MC 03	c 3246-5_3246-7del^a^	53	0	294.5	nd	15.03	nd	0.15	nd
NPC1-MC 04	c 3246-5_3246-7del^a^	61	5	341.7	481.67	18.06	28.03	0.10	0.12
NPC1-MC 05	c.3246-25A>G^b^	51	1	341	376.79	17.87	26.54	0.15	0.13
NPC1-MC 06	c.2861C>T (p.S954L)	61	0	298	477.26	17.45	28.69	0.15	0.16
NPC1-MC 07	c.2776G>A (p.A926T)	59	1	367	420.63	20.62	26.39	0.14	0.14
NPC1-MC 08	c.2861C>T (p.S954L)	54	1	296.7	351.74	21.14	28.41	0.17	0.18
NPC1-MC 09	c.2861C>T (p.S954L)	24	0	408	449.61	18.45	30.24	0.10	0.20
NPC1-MC 10	c.2861C>T (p.S954L)	61	2	571.6	628.54	20.59	28.97	0.09	0.10
NPC1-MC 11	c.2780C>T (p.A927V)	46	1	409.6	489.52	19.45	29.87	0.14	0.13
NPC1-MC 12	c.2474A>G (p.Y825C)	48	1	472	480.98	18.99	28.09	0.10	0.18
NPC1-MC 13	c.2978delG (p.G993fs)	39	2	383.7	416.66	18.31	31.12	0.14	0.25
NPC1-MC 14	c.1843C>T (p.R615C)	49	0	290.2	317.89	14.76	23.92	0.12	0.17
NPC1-MC 15	c.1211G>A (p.R404Q)	50	2	460.3	584.23	19.26	27.41	0.10	0.10
NPC1-MC 16	c.2974G>T (p.G992W)	53	nd	446.5	451.86	19.34	28.07	0.12	0.27
NPC1-MC 17	c.2974G>T (p.G992W)	20	0	479.3	nd	20.24	27.49	0.11	0.15

*NPC1-MC* NPC1-mutation carriers, *nd* no data, *N.y.f.* not yet found, *na* not applicable; motor score: 1 point each for presence of a reduced arm swing, intention tremor, increased muscle tone, ankle clonus, gait abnormalities; scores ≥ 1 were considered pathological; *mDRS* modified disability rating scale, *SARA* scale for the assessment and rating of ataxia, *PV* peak velocity, *SD* standard deviation; characteristics of vertical saccades in response to 20° stimulus and horizontal saccades in response to 30° are listed

^a^An intronic variant not previously described in the literature (class 3), leading to skipping of exon 22

^b^An intronic variant not previously described in the literature, likely pathogenic based on prediction analysis

### Optical coherence tomography (OCT)

OCT examination was performed using an SD-OCT (Spectralis, Heidelberg Engineering, Heidelberg, Germany) with automatic real-time (ART) function for image averaging. We acquired peripapillary retinal nerve fiber layer thickness (pRNFL), macular RNFL (mRNFL), and volumes of combined ganglion cell and inner plexiform layer (GCIP), and combined outer plexiform layer and outer nuclear layer (OPONL). All macular layers were calculated as a 3 mm-diameter cylinder around the fovea from a macular volume scan (20° × 20°, 25 vertical B-scans, ART ≤ 49). The peripapillary RNFL (pRNFL) was measured with activated eye tracker using 3.4 mm ring scans around the optic nerve (12°, 1536 A-scans, ART ≤ 100). Segmentation of all layers was performed semi-automatically using software provided by the OCT manufacturer (Eye Explorer 1.9.10.0 with viewing module 6.3.4.0, Heidelberg Engineering, Heidelberg, Germany). One experienced rater (JH), blinded for the diagnosis, carefully checked all scans for sufficient quality and segmentation errors and corrected if necessary. OCT data in this study are reported and analyzed according to the APOSTEL and OSCAR-IB recommendations [18, 19]. The data were correlated with VOG measures and clinical scores.

### Video-oculography (VOG)

Since patients with NPC demonstrate characteristic ocular motor deficits, including, but not limited to vertical supranuclear saccade palsy (VSSP), video-oculography (VOG) was performed in both NPC1-P and NPC1-MC, using a video-based eye-tracker system (EyeSeeCam^®^, Munich, Germany) as previously described [20, 21]. Briefly, reflexive saccades that reflect brainstem function and smooth pursuit for cerebellar function were assessed.

### Clinical assessment

To assess the overall neurologic status in NPC disease, the modified Disability Rating Scale (mDRS) by Pineda et al. [22] was applied; the mDRS is a four-domain scale (ambulation, manipulation, language, and swallowing) in an extended form [23], which also includes seizures and ocular movements, that assesses the severity of the disease and monitors the effect of treatment. All patients were examined by the same experienced neurologist (TBE) to reduce inter-rater variability. Cerebellar function was evaluated by administrating the Scale for the Rating and Assessment of Ataxia (SARA) [24] an eight-item clinical rating scale (gait, stance, sitting, speech, fine motor function, and taxis; range 0–40, with 0 being the best neurological status and 40 the worst). Moreover, NPC1-P were assessed by the Spinocerebellar Ataxia Functional Index (SCAFI), comprising the 8-m-Walking-Test (8MW), performed by having patients walking twice as quickly as possible from one line to another excluding turning, 9-Hole-Peg-Test (9HPT), and the number of “PATA” repetitions over 10 s (PATA) [25]. NPC1-MC were clinically assessed by an experienced neurologist in the field of movement disorders (SAS) with a particular focus on signs and symptoms of NPC disease: 1 point each was given for presence of a reduced arm swing, intention tremor, increased muscle tone, ankle clonus, and/or gait abnormalities resulting in a final motor score [26]. Scores ≥ 1 were considered pathological.

### Statistical analysis

The groups of patients and carriers were separately compared to a group of age-matched healthy controls. Data are presented as mean and standard deviation for continuous parameters and frequency and proportion for categorical variables. To compare OCT measures between subpopulations, and to consider within-patient inter-eye correlation, we used generalized estimation equation models (GEEs), in which the correlation matrix parameter was set to ‘exchangeable’. In these models, OCT measures were the dependent variables, while patient’s disease status was the (main) independent variable. Furthermore, in adjusted GEE analyses, the additional independent variables gender and age were added. To evaluate the strength of relationship between relevant OCT measures that were significantly decreased in homozygotes and clinical variables, we calculated the Spearmen´s correlation coefficients. Due to the fact that the correlations were not random but based on patient findings and were, therefore, limited in number, we did not perform a post-hoc correction. Statistical significance was achieved at *p* < 0.05. Data were analyzed with SAS version 9.3 (SAS institute, Cary, NC, USA) by a statistician (RS). Clinical correlations were performed in SPSS version 25.0.0 (IBM, Armonk, NY; done by TBE).

## Results

### Cross-sectional group differences

**Fig. 1:**
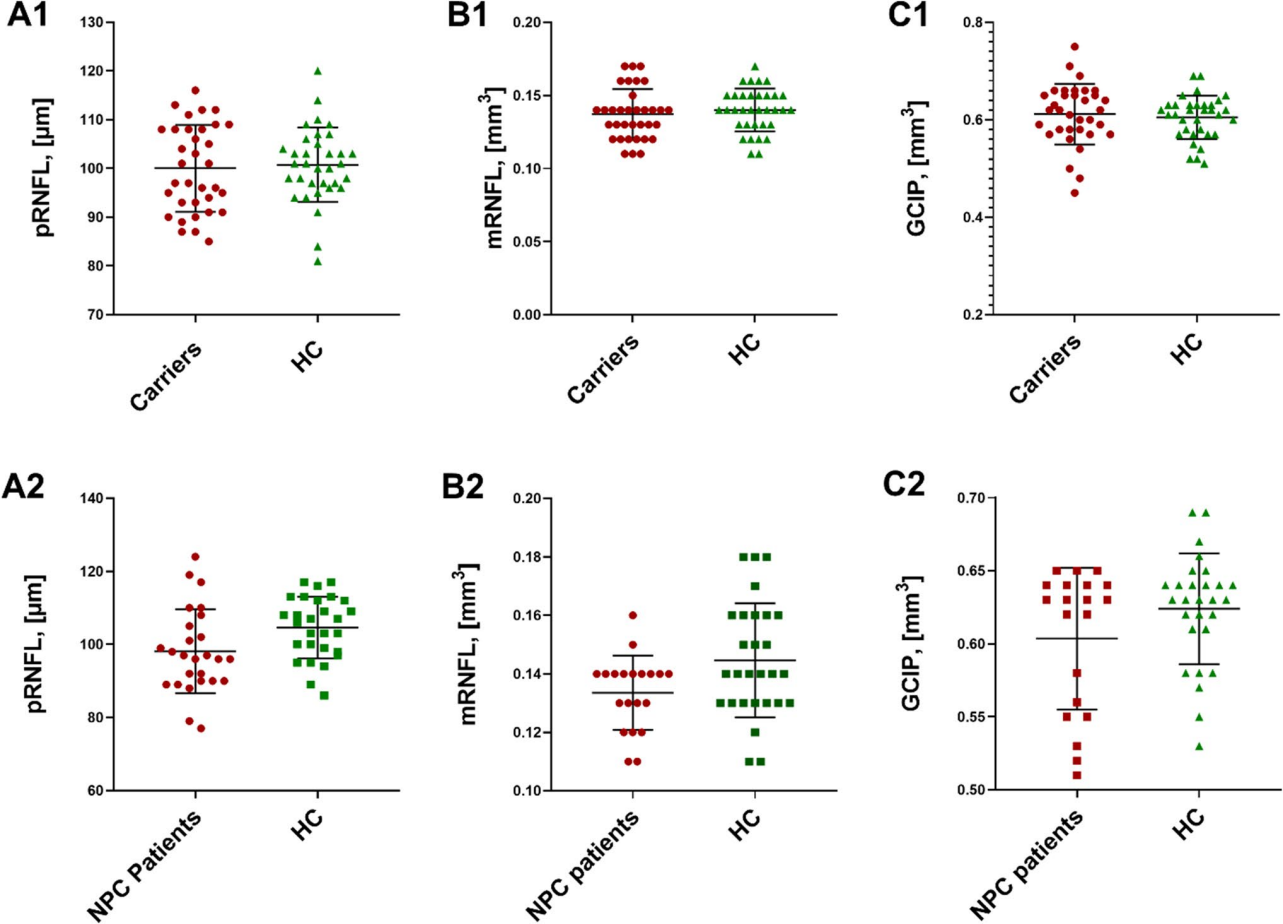
OCT results of NPC1-P, NPC1-MC, and HCs. NPC1-P: NPC1-Patients and NPC1-MC: NPC1-Mutation Carriers; Box plots of cross-sectional OCT data for NPC1-MC vs. HC (A1, B1, C1) and NPC1-P vs. HC (A2, B2, C2). A: peripapillary retinal nerve fiber layer (pRNFL), B: macular retinal nerve fiber layer (mRNFL) and C: ganglion cell and inner plexiform layer (GCIP)

For statistical analysis, we evaluated 26 eyes of 14 NPC1-P and 34 eyes of 17 NPC1-MC. These eyes were compared with 28 eyes of 14 age- and sex-matched HC. NPC1-P had a significantly lower mRNFL (mean ± SD, 0.13 ± 0.01 mm^3^) and GCIPL (0.60 ± 0.05 mm^3^) compared to HC (mRNFL 0.14 ± 0.02 mm^3^; *p* = 0.01; GCIP 0.62 ± 0.04 mm^3^; *p* = 0.04) (Fig. 1). pRNFL showed a trend, but the difference was non-significant (98.12 ± 11.46 µm vs. 104.61 ± 8.39 µm; *p* = 0.06). No significant differences were observed for PMB, TMV, and OPONL. Furthermore, there were no significant differences between NPC1-MC and HC for all studied parameters (summarized in Table 3). An orienting comparison of visual acuity data showed a reduced Snellen Visual Acuity Equivalent (SVAE) and a reduced number of recognized letters (100%/2.5% Low-Contrast Sloan Letter Chart Testing (2 m), see Table 3) in the group of NPC1-P. A statistical analysis was not performed for unequally distributed missing VA values.

**Table 3:** OCT results and visual data of NPC1-P, NPC1-MC, and HCs

	HC	NPC1-P	[*B*]	Significance [*p*]	HC	NPC1-MC	[*B*]	Significance [*p*]
pRNFL G [mean (SD) µm]	105 (8)	98 (11)	− 6.6964	0.0618	101 (8)	100 (9)	− 1.0669	0.6646
PMB [mean (SD) mm^3^]	56 (6)	54 (7)	− 2.3935	0.2454	55 (7)	54 (10)	− 1.5707	0.5660
TMV [mean (SD) mm^3^]	2.19 (0.10)	2.17 (0.12)	− 0.0438	0.2424	2.18 (0.08)	2.18 (0.10)	0.0020	0.9457
mRNFL [mean (SD) mm^3^]	0.14 (0.02)	0.13 (0.01)	− **0.0131**	**0.0116**	0.14 (0.01)	0.14 (0.02)	− 0.0028	0.5590
GCIP [mean (SD) mm^3^]	0.62 (0.04)	0.60 (0.05)	− **0.0306**	**0.0411**	0.61 (0.04)	0.61 (0.06)	0.0049	0.7670
OPONL [mean (SD) mm^3^]	0.67 (0.06)	0.66 (0.06)	− 0.0197	0.3754	0.66 (0.04)	0.66 (0.04)	− 0.0013	0.9194
Visual data available [% eyes]	50	68			65	94		
SVAE [mean dec, all eyes]	1.3	0.6	nd	nd	1.0	1.1	nd	nd
100% chart letters [mean N]	59	38	nd	nd	53	55	nd	nd
2.5% chart letters [mean N]	31	11	nd	nd	26	26	nd	nd

Mean and SD (standard deviation) in µm or mm^3^; *VA* visual acuity, *SVAE* Snellen visual acuity equivalent decimal; 100%/2.5% low-contrast Sloan letter chart testing (2 m) – *N* (number) of chart symbols (range 0–70); *nd* not done (statistical analysis was not performed due to unevenly distributed missing values)

*NPC1-P* NPC1-Patients, *NPC1-MC* NPC1-mutation carriers, *HC* healthy control, *OCT* optical coherence tomography, *B* estimate, *p* value, pRNFL peripapillary retinal nerve fiber layer, *PMB* papillo-macular bundle, *TMV* total macular volume, *mRNFL* macular retinal nerve fiber layer, *GCIP* combined ganglion cell and inner plexiform layer, *OPNL* outer plexiform layer and outer nuclear layer

### Correlation OCT with clinical scores

**Fig. 2:**
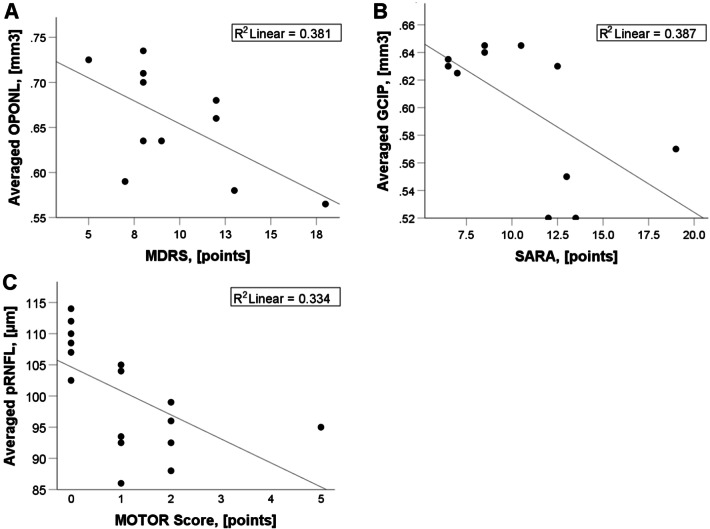
Relationships between OCT measures and disease severity scales in NPC1-P (**a**, **b**) and in NPC1-MC (**c**). *mDRS* modified disability rating scale, *SARA* scale for the assessment and rating of ataxia. Motor score: one point each for presence of a reduced arm swing, intention tremor, increased muscle tone, ankle clonus, and gait abnormalities. Scores ≥ 1 were considered pathological. R^2^ represents a goodness-of-fit measure for linear regression model. *pRNFL* peripapillary retinal nerve fiber layer thickness, *GCIP* volumes of combined ganglion cell and inner plexiform layer, *OPNL* combined outer plexiform layer and outer nuclear layer

In NPC1-P, mean mDRS score correlated only with mean OPONL (*ρ* = − 0.617, *p* < 0.05). Mean SARA score was negatively related to GCIP (*ρ* = − 0.622, *p* < 0.05) (Fig. 2a, b). In NPC1-MC, motor scores (Table 1) were negatively associated with mean pRNFL (Average: *ρ* = − 0.719; *p* < 0.01) (Fig. 2c). No further correlations with clinical scores were seen.

### Correlation OCT with saccades and smooth pursuit

**Fig. 3:**
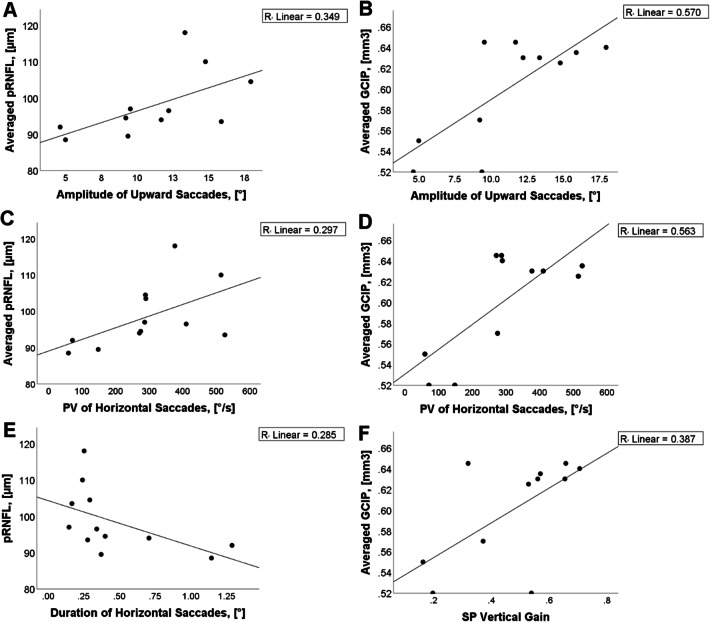
Relationships between OCT measures and video-oculography data. In **a** and **b** correlation with upward vertical saccades to 20° stimulus, in **c** and **d** with mean peak velocity (PV), in **e** with the duration of horizontal saccades, and in **f** with vertical smooth pursuit (SP) gain are shown. *pRNFL* peripapillary retinal nerve fiber layer thickness, *GCIP* volumes of combined ganglion cell and inner plexiform layer, *OPONL* combined outer plexiform layer and outer nuclear layer

Amplitude of upward vertical saccades to 20° stimulus in NPC1-P showed positive associations with averaged pRNFL (*ρ* = 0.645, *p* < 0.05) and averaged GCIP (*ρ* = 0.609, *p* < 0.05) (Table 1, Fig. 3a, b). Mean peak velocity of horizontal saccades to 30° stimulus correlated with averaged pRNLF (*ρ* = 0.650, *p* < 0.05) and averaged GCIP (*ρ* = 0.750, *p* < 0.01) (Table 1, Fig. 3c, d). Mean duration of horizontal saccades correlated negatively with pRNLF (*ρ* = − 0.727, *p* < 0.01) (Table 1, Fig. 3e). Averaged GCIP tracked with vertical smooth pursuit gain (*ρ* = 0.622, *p* < 0.05) (Table 1, Fig. 3f). In NPC1-MC, there were no significant associations between the retinal and video-ocular motor measures (data not shown).

## Discussion

In this study, we examined NPC1-P, clinically asymptomatic NPC1-MC, and HC using OCT to identify retinal degeneration and to evaluate the potential eligibility of OCT measures as biomarkers of neurodegeneration and disease progress. OCT proved to be a precise and reproducible method [27] for non-invasive visualization and quantification of retinal layers, and plays a crucial role in analyzing retinal changes in various neurodegenerative [15] and neuroinflammatory diseases [28–30]. Due to its anatomical, embryological, and physiological similarities with the brain, the retina offers a unique and easily accessible "window" to study correlates and consequences of subclinical pathology in the brain [31].

The major findings of this study are as follows: first, retinal neuronal and axonal degeneration in NPC1-P was significantly related to clinical disease scores. Second, for asymptomatic heterozygous NPC1-MC, there was no significant retinal degeneration. However, the extent of retinal neurodegeneration was significantly related to subclinical abnormalities in carriers.

In NPC1-P, we found a significantly lower thickness of mRNFL as well as a non-significant trend of reduced pRNFL compared to controls. The reduction of pRNFL has been described as a retinal marker of a possible dementia development, including AD [32], and may serve as a marker of disease progression in PD, AD, and frontotemporal dementia [33–35]. Our data are in line with those findings; however, only the reduction of mRNFL but not of the pRNFL reached the level of significance. The non-congruent effect of pRNFL and mRNFL might be due to the small volume of the RNFL measurement or a consequence of the small sample size. Our findings on reduced thickness of the retinal nerve fiber layer strengthen the hypothesis of a common pathomechanisms in NPC and other neurodegenerative diseases. pRNFL atrophy has also been reported in AD, PD, and other neurodegenerative diseases including rare diseases such as Leber’s hereditary optic neuropathy (LHON), OPA1-related dominant optic nerve atrophy (DOA) [36], Wilson`s disease [15], Friedreich`s ataxia [37], and spinocerebellar ataxias [38]. Histologic and OCT studies in AD and PD showed a significant loss of retinal ganglion cells [39, 40]. In line with these data, we found a significantly lower thickness of GCIP in NPC1-P compared to HC. However, the pathophysiology leading to retinal degeneration in NPC remains unclear. GCIP atrophy might be explained by the predominant mitochondrial dysfunction and oxidative stress [41]. In line with this hypothesis, functional studies in NPC have shown an increase in autophagy markers in the ganglion cell layer and the upregulation of proteins that mediate cellular cholesterol release in the retina [42]. Finally, inflammation has been suggested to play a key role in the pathogenesis of NPC [43]. In analogy to the known GCIP degeneration in multiple sclerosis [44], one might speculate that the atrophy of the ganglion cells in NPC1 could be at least be partly explained by inflammatory processes.

Recently, various subclinical abnormalities in asymptomatic heterozygote NPC1-mutation carriers have been described including hepatosplenomegaly, increased cholestantriol, and plasma chitotriosidase and features of early neurodegenerative disease, e.g., impaired cognitive function, hyposmia, features suggestive of REM sleep behavior disorder, and decreased glucose metabolic rates on PET imaging [26]. We found no retinal neuronal and axonal degeneration in our NPC1-MC, but we did observe a non-significant trend towards lower mRNFL as seen in NPC1-P. Notably, peripapillary retinal nerve fiber layer thickness in NPC1-MC correlated with suspicious motor scale scores. These preliminary observations are of special interest, as they are in line with OCT findings in a related metabolic disorder, Gaucher’s disease (GD), in which heterozygous mutations in the GBA gene are the most frequent genetic risk factor for developing late-onset Parkinson’s disease [45]. In GD ganglion cell layer atrophy was found in GD patients but also in asymptomatic mutation carriers [17]. Indeed, animal studies in NPC1-mutant mice demonstrated preclinical retinal degeneration and lipofuscin accumulation in the pigment epithelium and ganglion cells, electrodense inclusions in various cell types, photoreceptor defects, and hyperactivity of glial cells [42, 46], as well as optic nerve pallor and per macular grey discoloration [47]. Given the pathomechanistic similarities between NPC and GD, we encourage additional work in NPC heterozygotes to shed further light on potential similar risks.

As we did observe a correlation between OCT values and subtle neurologic findings in clinical tests and scores in patients and in heterozygous mutation carriers, we were able to evaluate the potential eligibility of OCT measures as biomarkers of neurodegeneration and disease progress. In NPC1-P, a clear negative correlation between a decrease in GCIP as a sign of retinal neurodegeneration and an increase in cerebellar ataxia as a sign of severity of the NPC1-phenotype could be shown. In addition, the extent of the decrease in OPONL correlated with the severity of motor impairment (mean mDRS). Furthermore, we correlated saccadic and smooth pursuit measures with retinal characteristics. The amplitude of upward vertical saccades to 20° stimulus and the mean peak velocity of horizontal saccades to 30° stimulus in NPC1-patients showed positive associations with averaged pRNFL and averaged GCIP. In NPC1-MC, there were no significant associations between the retinal and ocular motor measures. In line with the VOG study of a large NPC cohort [20], horizontal saccades peak velocity and duration are related to the disease characteristics. This is due to the functional impairment of burst neurons in rostral interstitial nucleus of the medial longitudinal fascicle (riMLF) in the rostral mesencephalon. Based on the correlations shown, it might be considered to monitor disease progression in NPC1-P after clinical manifestation by measuring retinal axonal and neuronal degeneration.

The study has several limitations. Due to the rarity of this metabolic disease, the sample size is small and confirmation by an independent group is encouraged. We took a cross-sectional approach, but future longitudinal studies may help to validate the described retinal pattern. In addition, NPC1-P with the presumably most pronounced retinal pathology could not be included in the study. Due to their severe clinical affection such as pronounced cerebellar ataxia and dementia, they had difficulties to undergo OCT.

In summary, by retinal imaging using the OCT technique, we provided additional and complementary diagnostic information in symptomatic NPC1-P. Since OCT is a non-invasive, safe, and highly precise diagnostic tool, we expect an increasing impact of OCT in the diagnostic work-up of NPC, particularly of adult-onset NPC1, in which the clinical symptoms are often mild and non-specific. Furthermore, longitudinal OCT analyses will help to understand the disease specificity of our findings and the opportunities to integrate this technique into the daily clinical practice. Finally, on the basis of our findings, the role of OCT to monitor disease progression and treatment response in NPC1 has to be evaluated.
